# Analysis of mass spectrometry data from the secretome of an explant model of articular cartilage exposed to pro-inflammatory and anti-inflammatory stimuli using machine learning

**DOI:** 10.1186/1471-2474-14-349

**Published:** 2013-12-13

**Authors:** Anna L Swan, Kirsty L Hillier, Julia R Smith, David Allaway, Susan Liddell, Jaume Bacardit, Ali Mobasheri

**Affiliations:** 1 School of Biosciences, Faculty of Science, University of Nottingham, Sutton Bonington Campus, Leicestershire, LE12 5RD, UK; 2 Musculoskeletal Research Group, School of Veterinary Medicine and Science, Faculty of Medicine and Health Science, University of Nottingham, Sutton Bonington Campus, Leicestershire, LE12 5RD, UK; 3 Bruker UK Limited, Coventry, CV4 9GH, UK; 4 WALTHAM® Centre for Pet Nutrition, Waltham-on-the-Wolds, Melton Mowbray, Leicestershire, LE14 4RT, UK; 5 Proteomics Laboratory, School of Biosciences, University of Nottingham, Sutton Bonington Campus, Leicestershire, LE12 5RD, UK; 6 School of Computer Science, University of Nottingham, Jubilee Campus, Nottingham, NG8 1BB, UK; 7 The D-BOARD European Consortium for Biomarker Discovery, University of Nottingham, University Park, Nottingham, NG7 2RD, UK; 8 School of Computing Science, Newcastle University, Claremont Tower, Newcastle-upon-Tyne, NE1 7RU, UK; 9 Arthritis Research UK Centre for Sport, Exercise and Osteoarthritis, Nottingham University Hospitals, Nottingham, NG7 2UH, UK; 10 Arthritis Research UK Pain Centre, The University of Nottingham, Queen's Medical Centre, Nottingham, NG7 2UH, UK; 11 Medical Research Council and Arthritis Research UK Centre for Musculoskeletal Ageing Research, The University of Nottingham, Queen’s Medical Centre, Nottingham, NG7 2UH, UK; 12 Center of Excellence in Genomic Medicine Research (CEGMR), King Fahad Medical Research Center (KFMRC), King AbdulAziz University, Jeddah, 21589, Kingdom of Saudi Arabia; 13 Schools of Pharmacy and Life Sciences, University of Bradford, Richmond Road, Bradford, BD7 1DP, UK; 14 Comparative Physiology, Medical Research Council-Arthritis Research UK Centre for Musculoskeletal Ageing Research, Arthritis Research UK Pain Centre, Arthritis Research UK Centre for Sport, Exercise, and Osteoarthritis, Faculty of Medicine and Health Sciences, The University of Nottingham, Sutton Bonington Campus, Leicestershire, LE12 5RD, UK; 15 Faculty of Medicine and Health Sciences, The University of Nottingham, Sutton Bonington Campus, Leicestershire, LE12 5RD, UK

**Keywords:** Osteoarthritis, Cartilage, Biomarker, Interleukin 1 β, Carprofen, Bioinformatics, Machine learning

## Abstract

**Background:**

Osteoarthritis (OA) is an inflammatory disease of synovial joints involving the loss and degeneration of articular cartilage. The gold standard for evaluating cartilage loss in OA is the measurement of joint space width on standard radiographs. However, in most cases the diagnosis is made well after the onset of the disease, when the symptoms are well established. Identification of early biomarkers of OA can facilitate earlier diagnosis, improve disease monitoring and predict responses to therapeutic interventions.

**Methods:**

This study describes the bioinformatic analysis of data generated from high throughput proteomics for identification of potential biomarkers of OA. The mass spectrometry data was generated using a canine explant model of articular cartilage treated with the pro-inflammatory cytokine interleukin 1 β (IL-1β). The bioinformatics analysis involved the application of machine learning and network analysis to the proteomic mass spectrometry data. A rule based machine learning technique, BioHEL, was used to create a model that classified the samples into their relevant treatment groups by identifying those proteins that separated samples into their respective groups. The proteins identified were considered to be potential biomarkers. Protein networks were also generated; from these networks, proteins pivotal to the classification were identified.

**Results:**

BioHEL correctly classified eighteen out of twenty-three samples, giving a classification accuracy of 78.3% for the dataset. The dataset included the four classes of control, IL-1β, carprofen, and IL-1β and carprofen together. This exceeded the other machine learners that were used for a comparison, on the same dataset, with the exception of another rule-based method, JRip, which performed equally well. The proteins that were most frequently used in rules generated by BioHEL were found to include a number of relevant proteins including matrix metalloproteinase 3, interleukin 8 and matrix gla protein.

**Conclusions:**

Using this protocol, combining an *in vitro* model of OA with bioinformatics analysis, a number of relevant extracellular matrix proteins were identified, thereby supporting the application of these bioinformatics tools for analysis of proteomic data from *in vitro* models of cartilage degradation.

## Background

Articular cartilage is a mechanically resilient connective tissue with unique load-bearing and shock-absorbing properties, which are largely dependent on the structural and functional integrity of its highly charged and hydrated extracellular matrix (ECM) [1]. Cartilage contains three principal components: chondrocytes, aggregating proteoglycans and collagens, all of which are embedded within the ECM and contribute to the homeostasis of the tissue [2]. Cartilage relies on oxygen and nutrient delivery from the synovial fluid [3] but is avascular and recalcitrant to repair [4]. Osteoarthritis (OA) is a degenerative disease of synovial joints, involving the loss of articular cartilage, synovial inflammation and changes to the subchondral bone, resulting in impaired articulation, reduced mobility, joint stiffness and pain [5,6]. OA is estimated to affect up to 85% of the human population over 60 years old [7] and is also common in companion animals [8]. There are a number of factors affecting OA, including age, obesity, previous joint trauma or instability, metabolic or endocrine disease and oestrogen status [9,10]. Currently, diagnosis is made through clinical examination and the imaging “gold standard”, radiography. However, radiographic diagnosis of OA is usually made when the clinical signs of pain and loss of mobility have already appeared. Consequently, the disease can remain undiagnosed until the later stages, where interventions may not alter the course of progression.

Biomarkers have the capacity to identify early changes in joint tissues and diagnose OA during the pre-radiographic stages of the disease and to determine the course of its progression, as well as aid in drug discovery and clinical trials [11-15]. The term biomarker can be used to describe molecules or molecular fragments that indicate the presence of a biological or disease process. Early detection may also help prioritize treatments to slow progression, such as weight loss and a reduction in high impact load bearing on those joints [16]. Therefore, individual or combination biomarkers must be able to clearly differentiate between healthy and diseased states. Ideally biomarkers should be disease-specific and not be influenced by other disorders. Biomarkers should also be easily measurable in a clinical setting [17]. In rheumatology, biomarkers can be “tissue fingerprints” or combinations of “neo-epitopes”, reflecting catabolic effects downstream of inflammatory signals.

Recent advances in post-genomic technologies, including genomics, transcriptomics, proteomics and metabolomics, have allowed the development of novel methods for identification of biomarkers of disease. Proteomics is a particularly promising technology as it allows the identification of individual proteins and their peptides, neo-epitopes and degradation “fingerprints”. This information can then be used to develop sensitive, rapid antibody-based assays. In addition proteomic analyses provide an overview of changes in the proteome in biological systems across a range of conditions [18].

Through the combined use of proteomics, transcriptomics and other biochemical and immunological techniques, a number of proteins and protein families have previously been associated with OA. These include ECM proteins such as aggrecan, the major structural proteoglycan found in the cartilage ECM, cartilage oligomeric matrix protein (COMP), a non-collagenous protein involved in the organization and assembly of articular cartilage, and matrix metalloproteinases (MMPs), a family of proteins expressed by chondrocytes, which are involved in the degradation of ECM macromolecules and lead to the fibrillation of articular cartilage [11,19-24]. In the ECM, matrix metalloproteinase-3 (MMP-3) in particular appears to be vital for matrix turnover and homeostasis. This protein is up-regulated in early OA, but has been found to be down-regulated in later stages of the disease [25].

Many omics technologies, such as microarrays, next generation sequencing and mass spectrometry (MS), generate large amounts of data. Therefore, bioinformatic tools play an important role in the analysis of such data and a wide range of methods have been developed for this purpose [26,27]. Supervised machine learning techniques are used, based on a training set of labelled samples, to build models that are able to automatically label previously unclassified samples [28,29]. Samples can be assigned a label (e.g. a treatment group) based on whether or not they contain a certain attribute (e.g. a protein, or a group of proteins) and at what level the attribute is found within the samples [30,31]. There are many types of machine learning techniques, such as decision trees, rule-based learners and support vector machines [28,32]. Rule-based machine learning methods automatically produce human-readable production rules that assign samples to their respective treatment groups. In proteomics-based approaches, the rules created contain proteins that best divide the samples into disease or treatment groups. Proteins most consistently differing between groups are suitable for further investigation as potential biomarkers.

The aim of this study was to identify suitable bioinformatic methods for the analysis of proteomics data generated to investigate cytokine-induced catabolic changes associated with the early stages of OA [33]. This involved using an explant model of cartilage to investigate the secretome of canine articular cartilage. The cartilage explant model was selected because it allows a rapid and ‘clean’ analysis of secreted proteins in the context of joint disease. Many of the proteins present in the secretome of explant cultures are involved in the control of physiological and pathophysiological processes in the joint [34] and may enter the blood stream where they may be accessible as systemic biomarkers.

## Methods

### Animal tissues and statement of ethical approval

Forelimbs and hind limbs were taken from male German Shepherd army dogs, over 5 years of age, that were euthanized for clinical reasons unrelated to research. Therefore, this project does not fall under the Animals (Scientific Procedures) Act 1986^a^ or the Veterinary Surgeons Act 1966^b^. Approval for the use of clinical materials was obtained from the Ethics Committee of the School of Veterinary Science and Medicine with input from members of the University of Nottingham's Animal Welfare and Ethical Review Body (AWERB). The British Army owned the animals that were used in this study. Informed consent was obtained for the use of joint tissues.

### Cartilage explant culture

Limbs were washed in disinfectant and soaked in sodium hypochlorite prior to spraying with ethanol. The stifle and elbow joints were dissected under sterile conditions and full thickness articular cartilage was placed in serum free collection media. The media consisted of Hyclone^®^ liquid medium: DMEM supplemented with penicillin and streptomycin.

After washing the harvested cartilage, a 3 mm biopsy punch was used to cut discs, which were placed in a randomized manner into wells of a 24 well plate, containing serum free DMEM (as above). The media was removed and the explants were incubated in media alone (control), or supplemented with recombinant canine IL-1β (10 ng/ml), the non-steroidal anti-inflammatory drug carprofen (Rimadyl^®^, 100 μg/ml), or carprofen and IL-1β combined (100 μg/ml and 10 ng/ml, respectively). For each treatment, three samples were used per dog, giving six samples per treatment. After 5 days in culture, supernatants and explants were removed and processed for mass spectrometric analysis.

### Sample preparation and mass spectrometry

Samples from 2 dogs were chosen for MS/MS analysis based on the general profile of proteins as visualized on SDS-PAGE (data not included in the manuscript, see Additional file 1: Table S1; Additional file 2: Table S2; Additional file 3: Table S3; Additional file 4: Table S4; Additional file 5: Figure S1; Additional file 6: Figure S2). Each set of dog samples consisted of three treatments (IL-1β, carprofen, IL-1β + carprofen), with three replicates for each treatment for both dogs. A set of control samples was also analyzed, providing a total of 24 samples (12 samples per dog).

The secretome samples were digested with trypsin before mass spectrometry. Soluble proteins were reduced by the addition of DTT to a final concentration of 10 mM to each sample. The thiol groups were blocked by the addition of iodoacetamide to a final concentration of 55 mM. The proteins were then precipitated with ice-cold acetone before being suspended in trypsin solution (10 ng/μl in 50 mM ammonium bicarbonate) (Trypsin Gold, Mass Spectrometry Grade, Promega). Trypsin digestion was terminated by addition of formic acid to give a final concentration of 0.1%. Before MS analysis, an aliquot of the digestion was desalted and any insoluble particulates removed using a C18 Zip-Tip (Millipore).

Peptides were separated on a 15 cm C18 PepMap™ column (LC Packings) using a Bruker Easy-nLC platform with a flow rate of 300 nl/min. The sample was added to solvent A (95% v/v H_2_O, 5% v/v ACN, 0.1% v/v formic acid) and was injected into the HPLC column via the autosampler. Following binding and washing of the sample on the column in solvent A, peptides were separated and eluted in a gradient of solvent B (95% v/v ACN, 5% v/v H_2_O, 0.1% v/v formic acid).

Eluted peptides were delivered on-line and detected in a Bruker AmaZon ETD ion trap instrument. The five most abundant peptides in each MS scan were selected for fragmentation. The raw data were processed to provide peptide and fragment mass lists which were submitted to the MS/MS ions tool of the Mascot search engine, software which uses protein sequence databases to predict the identity of proteins present in samples, based on the peptides identified. The fragment mass values for each peptide were compared to the mammalian entries from the UniProtKB database. The modifications incorporated into the search were: fixed carbamidomethyl cysteine and variable oxidation of methionine.

One sample, treated with both IL-1β + carprofen, was removed from the dataset at this stage as it was considered to be anomalous due to the very small number of proteins that were identified from it by Mascot. This resulted in 23 samples for further analysis: six samples per treatment, except for IL-1β + carprofen, for which there were five samples.

### Further MS data analysis pipeline

**Figure 1:**
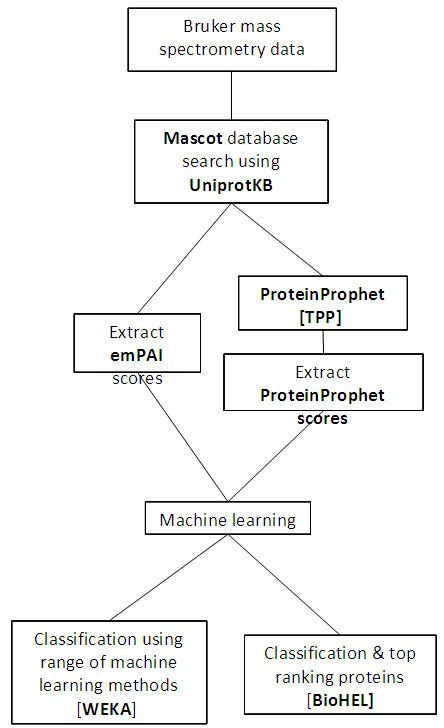
**Pipeline for label-free quantification of mass spectrometry data.** TPP – stages included in the Trans-Proteomic Pipeline.

The pipeline for the analysis of mass spectrometry data is described in Figure 1. Included in the results generated by Mascot is the exponentially modified protein abundance index (emPAI) score for each protein identified. The emPAI score gives an estimate for the absolute amount of a protein present in a sample [35]. It is based on the protein abundance index (PAI), which is defined as ‘the number of peptides identified divided by the number of theoretically observable tryptic peptides’ [36]. PAI was then adapted to emPAI to ensure it is proportional to the total protein content in a sample [35].

Mascot outputs were also submitted to ProteinProphet [37,38], part of the Trans-Proteomic Pipeline [39], used for the statistical validation of protein identifications. Using ProteinProphet a probability score is assigned to each of the protein identifications that was made by Mascot. Strictly speaking, ProteinProphet is not a true quantification method, but the probability scores that it produces are roughly equivalent to a quantitative approach. Therefore it is suitable for further analysis using machine learning techniques.

Both the emPAI and ProteinProphet scores were generated. Machine learning was applied to these datasets using a number of methods from the WEKA machine learning package [32] and BioHEL, a rule based learner [31].

### Comparison of machine learning techniques

To determine the most suitable machine learning method for the analysis of canine articular cartilage mass spectrometry dataset, seven different machine-learning techniques, including BioHEL, were applied to compare their abilities. The other methods used were Naive Bayes, Support Vector Machines, C4.5, IBk, JRip and Random Forest, all implemented in WEKA. The source code and user manual for BioHEL are available at http://ico2s.org/software/biohel.html.

Due to some anomalous identifications in the Mascot results for one of the carprofen + IL-1β samples, where only a very small number of proteins were identified compared to the other samples, it was removed from the dataset. This resulted in a dataset of 23 samples, spanning four treatment classes. As a result of this small number of samples, leave-one-out cross validation was used to divide them into training and test sets [40]; using this method allows for the most information to be extracted from the data available. Twenty-three training sets and the same number of test sets were created. The test sets each contained only one sample, with the remainder of the dataset in the related training set. This allows the ability of classification models to be evaluated.

### Significance testing

The significance of the BioHEL classification accuracies achieved was tested by calculating *p*-values using one-tailed permutation testing [41]. A new version of the dataset was created where the samples were randomly assigned to treatments, but maintaining the same number of samples per treatment as in the original data. Afterwards, BioHEL was run, using leave-one-out cross-validation, to compare the accuracies achieved; 50 such permutations were generated for the emPAI, ProteinProphet and combined datasets. The accuracies achieved by these runs were compared to the accuracies achieved on the real, non-randomized, datasets and a *p*-value of the likelihood that the accuracy on the original data belongs to the randomized distribution was computed.

#### ***Identification of top ranking proteins***

Due to the performance of BioHEL in the comparison with other machine learning methods, analyses using BioHEL continued through the identification of proteins that were pivotal to the classification, using a methodology previously used for the analysis of transcriptomics data [42,43].

BioHEL classifies samples by automatically producing rules sets that consist of a number of rules that use the proteins found in the samples to determine which treatment group they belong to. Each rule within a set uses proteins, when used with mass spectrometry data, to assign samples to treatment classes. A rule within a set uses one or more proteins and assigns samples to the relevant class, shown at the end of rule, if it matches exactly the protein content specified by the rule. An example of a rule set for this data follows:

1. **If** the abundance of TPIS is greater than 0.01 **then** the sample belongs to the IL-1β group

2. **If** the abundance of IL-8 is greater than 0.02 **then** the sample belongs to the carprofen+IL-1β group

3. **If** the abundance of MMP-3 is greater than 0 **and** the abundance of UBIB is less than 0.2 **then** the sample belongs to the IL-1β group

4. **If** the abundance of MGP is greater than 0 **and** the abundance of A1AT is less than 0.9 **then** the sample belongs to the carprofen group

5. **If** the abundance of ALBU is greater than 0.01 **then** the sample belongs to the carprofen+IL-1β group

6. Any sample not assigned to a group belongs to the control group

The combinations of rules in the rule sets are used to assign samples to their respective treatment groups. Each rule contains one or more proteins and a score (either emPAI or ProteinProphet), which each protein should either be above or below, depending on the sign used. At the end of each line is the treatment class to which each rule relates. For example, the 1^st^ rule of the rule set shown classifies all samples as belonging to the IL-1β class if the value of the protein attribute TPIS is greater than 0.01. There are no rules for the control: all samples that are not assigned to the other three classes by the rules generated will be, by default, considered as a control sample.

Due to the stochastic nature of BioHEL, running it multiple times on the same dataset produces different rule sets. Therefore BioHEL was run 10,000 times to analyze the results and determine recurrent patterns. Proteins were ranked by the number of times they appeared in rules across the 10,000 runs, to highlight those proteins used most frequently. Those ranking at the top are proteins that can be used to most successfully identify between samples of different treatments. As these proteins are the most different between treatment classes, they may be suitable for consideration as biomarkers or further analysis of them may provide information about possible novel methods for diagnosis or treatment.

#### ***Network generation***

To investigate interactions between proteins with our prediction model we used network analysis, by identifying proteins that were working together in rules generated by BioHEL. Within rules generated by BioHEL protein pairs can be identified, from which networks were generated. These networks can be used to identify relationships between proteins; they also provide a visual way of viewing those proteins that are frequently in rules through identification of the most connected proteins. In the example of a rule set, shown in “***Identification of top ranking proteins*** ***” subsection***, there are some rules that use more than one protein; these were used to form protein pairs. For example, in the third rule both apolipoprotein E (APOE) and hyaluronan and proteoglycan link protein 1 (HPLN1) are used and so are considered a protein pair. The 100 protein pairs that were most frequently used within rules, for each individual treatment class, across the 10,000 runs of BioHEL were extracted and a network was generated from them in Cytoscape [44]. The networks consist of nodes that relate to the proteins, found in the BioHEL rules, and edges connect proteins if they were frequently included in rules together. The edges were then coloured based on the treatment class that each pair of proteins relates to.

## Results and discussion

Proteomic techniques are increasingly being used for the identification of novel joint disease biomarkers [11,19,20,45]. This study tests the hypothesis that the secretome of canine articular cartilage may provide a simple but well-defined model for studying potential biomarkers of early cartilage damage. To study the secretome of canine articular cartilage in an explant model we used a combination of conventional and high throughput proteomic techniques, followed by the application of bioinformatics techniques.

Although the cartilage explant system has not been used extensively in proteomic studies, a similar equine explant model of articular cartilage has been used to examine changes in the secretome in response to pro-inflammatory and anti-inflammatory stimuli [33]. This present study indicates that canine cartilage explants can also serve as a model for targeted and high throughput proteomic studies. This is supported by the identification of a large number of proteins whose functions are relevant to articular cartilage and biological processes that are relevant to joint disease and OA. Using the explant model this study has demonstrated it is feasible to incorporate pathophysiologically relevant stimuli such as pro-inflammatory cytokines (e.g. IL-1β) to simulate catabolic changes as well as NSAIDs (e.g. carprofen) to simulate pharmacotherapy in a well-controlled model *in vitro.*

The SDS-PAGE protein profiles of the IL-1β stimulated samples illustrate that some proteins are present at a higher level of abundance in the presence of IL-1β. This was demonstrated by the presence of extra bands in the IL-1β treated samples that were not detected in the controls (see Additional file 1: Table S1; Additional file 2: Table S2; Additional file 3: Table S3; Additional file 4: Table S4; Additional file 5: Figure S1; Additional file 6: Figure S2). There was also general consistency in protein profiles across all groups of treated samples for the two animals (see Additional file 1: Table S1; Additional file 2: Table S2; Additional file 3: Table S3; Additional file 4: Table S4; Additional file 5: Figure S1; Additional file 6: Figure S2).

**Figure 2:**
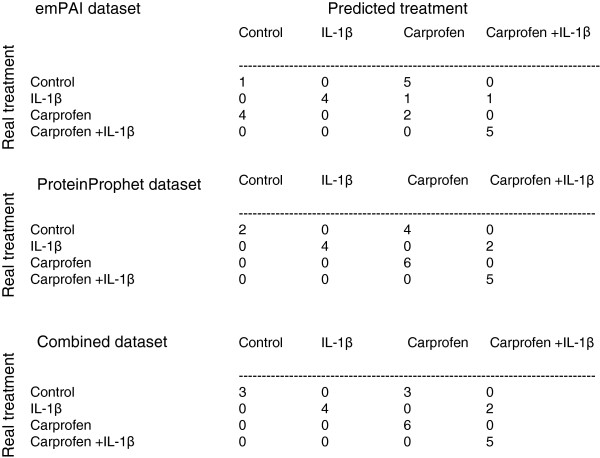
Confusion matrices, for the emPAI, ProteinProphet and combined datasets, to show the number of samples in each class and which class they were predicted to be in, using BioHEL.

A range of machine learning methods were compared and BioHEL proved to be successful in classifying both the ProteinProphet and emPAI datasets. The accuracies of the range of machine learning techniques tested on the canine articular cartilage data are shown in Table 1. For the BioHEL classifications on each dataset, confusion matrices (that identify, treatment by treatment, how the samples were predicted) were generated to understand which samples were predicted correctly. It can be seen from the matrices for the emPAI, ProteinProphet and combined datasets (Figure 2) that the most frequent incorrect prediction made was predicting control samples as carprofen treated samples. This is due to the similarity between these groups, as carprofen was added in the absence of IL-1β and thus there was no pro-inflammatory present for this NSAID to act on. No IL-1β sample was predicted as a control sample. From Table 1, it can be seen that BioHEL achieves the highest accuracies for both the ProteinProphet and the dataset that combines both emPAI and ProteinProphet scores; because of this, analysis was continued using BioHEL. The classification was increased by the combination of these two scoring systems. The significance of the BioHEL classification accuracies was supported by the *p*-values, calculated using permutation testing, shown in Table 2, as they were all close to zero. The outcome of this test confirms that the models generated by BioHEL (rule sets) are sound and hence we can safely analyze them to extract rankings of important variables and generate interaction networks.

**Table 1:** Comparison of performance accuracies, as percentage of samples correctly classified, for classification of canine articular cartilage data for seven different machine-learning methods, using leave-one-out cross-validation

**Dataseter**	**Naive Bayes**	**SVM**	**k-nearest neighbour**	**JRip (rule based)**	**Random forest**	**C4.5**	**BioHEL**
ProteinProphet	39.1	52.2	34.8	43.5	34.8	52.2	**73.9**
emPAI	52.2	56.5	52.2	**78.3**	39.1	73.9	56.5
ProteinProphet and emPAI combined	52.2	52.2	43.5	**78.3**	26.1	47.8	**78.3**

For the ‘ProteinProphet and emPAI combined’ the two scores were combined into one dataset. The highest accuracies achieved in each dataset are shown in bold.

**Table 2:** ***P*-values generated by significance testing of BioHEL for the emPAI, ProteinProphet and combined datasets**

**Dataset**	***P*-value**
emPAI	1.64E-100
ProteinProphet	1.23E-220
ProteinProphet and emPAI combined	0

*P*-values were generated using a one-tailed permutation test. Classifications were run on datasets with randomized sample labels, using leave-one-out cross-validation; this was performed 50 times.

From the rules generated by BioHEL, the top ranking mammalian proteins for the three treatments are shown in Tables 3 and 4. There is no ranking for the control class because it was used as the default and so did not include any proteins in rules. The default is included at the end of a rule set, so that any sample that has not been assigned to a class by the rules in the set is automatically placed into the default class. Table 5 shows the top ranking mammalian proteins for the emPAI and ProteinProphet combined datasets. It shows that both the emPAI and ProteinProphet scores were useful in the classification as some proteins, including triosephosphate isomerase, MMP-3, IL-8 and HPLN1, are top ranking proteins using both emPAI and ProteinProphet values.

**Table 3:** The ten mammalian proteins found most frequently in rules for each of the three classes, not including the default control class, from the ProteinProphet dataset

**IL-1β**	**Carprofen**	**IL-1β and Carprofen**
Matrix metalloproteinase 3 (47.5)	Matrix gla protein (52.5)	interleukin 8 (93.1)
Triosephosphate isomerase (19.6)	Apolipoprotein E (44.8)	Matrix metalloproteinase 3 (6.87)
Enolase (4.56)	Hyaluronan and proteoglycan link protein 1 (22.7)	Pyruvate kinase isozyme M1 (5.59)
interleukin 8 (2.61)	Target of Nesh-SH3 (5.24)	Protein S100-A1 (5.39)
Leukocyte antigen CD37 (2.58)	Phosphatidylinositol 3,4,5-trisphosphate 3-phosphatase and dual-specificity protein phosphatase PTEN (5.1)	Bardet-Biedl syndrome 10 protein (5.35)
Fibromodulin (2.58)	Extracellular matrix protein 1 (4.84)	50S ribosomal protein L29 (5.26)
Hyaluronan and proteoglycan link protein 1 (1.79)	Alpha-1-antitrypsin (2.89)	Max-like protein X (5.08)
Cartilage intermediate layer protein 1 (1.32)	Keratin (2.82)	Trypsin (4.82)
Metalloproteinase inhibitor 1 (1.22)	Decorin (2.59)	Triosephosphate isomerase (4.74)
Thrombospondin 1(1.13)	Myotubularin-related protein 1 (2.54)	Alpha-2-HS-glycoprotein (4.62)

The percentage of rules each protein was used in is shown in brackets.

**Table 4:** The ten mammalian proteins found most frequently in rules for each of the three classes, not including the default control class, from the emPAI dataset

**IL-1β**	**Carprofen**	**IL-1β and Carprofen**
Triosephosphate isomerase (73.4)	Alpha-2-HS-glycoprotein (99.3)	interleukin 8 (81.4)
Albumin (40.4)	Tumor necrosis factor receptor superfamily member 11B (15.6)	Lumican (14.9)
Serum amyloid A protein (23)	Pseudouridylate synthase 7 homolog (15.1)	Matrix metalloproteinase 3 (3.1)
Matrix metalloproteinase 3 (21.3)	Lysozyme C (2)	Desmin (2.6)
Vimentin-1 (9.8)	Apolipoprotein E (1.8)	Clusterin (2)
Vimentin (6)	Matrix gla protein (1.8)	Syndecan-4 (1.4)
Enolase B (5.9)	Fibromodulin (1.4)	Ribonuclease 4 (1)
Thrombospondin 1(5.3)	Clusterin (1.3)	Thrombospondin-3 (0.8)
Enolase A (3.4)	Metalloproteinase inhibitor 1 (0.6)	Enolase (0.6)
Keratin (3.8)	Retinoblastoma-like protein 2 (0.4)	Cartilage intermediate layer protein 1 (0.4)

The percentage of rules each protein was used in is shown in brackets.

**Table 5:** The ten mammalian proteins found most frequently in rules for each of the three classes, not including the default control class, from the ProteinProphet and emPAI combined dataset

**IL-1β**		**Carprofen**		**IL-1β and Carprofen**	
Triosephosphate isomerase (19.8)	PP	Apolipoprotein E (13.7)	PP	interleukin 8 (33.8)	emPAI
Albumin (10)	emPAI	interleukin 8 (10.8)	emPAI	interleukin 8 (31)	PP
Triosephosphate isomerase (9.3)	emPAI	interleukin 8 (9.9)	PP	Clusterin (5.8)	emPAI
Matrix metalloproteinase 3 (7.6)	PP	Matrix gla protein (9.3)	PP	Matrix metalloproteinase 3 (5.7)	PP
Thrombospondin 1 (6.2)	PP	Hyaluronan and proteoglycan link protein 1 (8.4)	PP	Thrombospondin-3 (2.4)	emPAI
Serum amyloid A protein (3.5)	emPAI	Fibromodulin (7.4)	emPAI	Matrix metalloproteinase 3 (2.2)	emPAI
Matrix metalloproteinase 3 (3.4)	emPAI	Transmembrane protein PVRIG (4.6)	PP	Ribonuclease 4 (1.9)	PP
Enolase B (3)	emPAI	Matrix gla protein (3.2)	emPAI	Ribonuclease 4 (1.7)	emPAI
Keratin (2.5)	emPAI	Hyaluronan and proteoglycan link protein 1 (2.3)	emPAI	Cartilage intermediate layer protein 1 (1)	emPAI
Enolase A (2.4)	emPAI	Clusterin (2)	emPAI	Lumican (1)	emPAI

The percentage of rules each protein was used in is shown in brackets. Alongside the proteins the table reports whether the rules were using the protein with its ProteinProphet probability (PP) or emPAI score.

**Figure 3:**
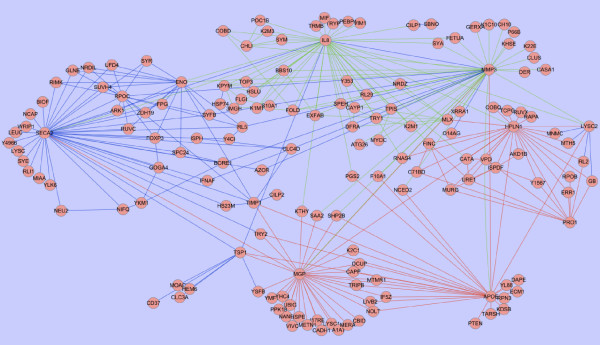
**Protein interaction network generated from the top 100 BioHEL protein pairs for the ProteinProphet canine articular cartilage dataset.** The most frequently used protein pairs for IL-1β in blue, carprofen in red and carprofen + IL-1β in green.

**Figure 4:**
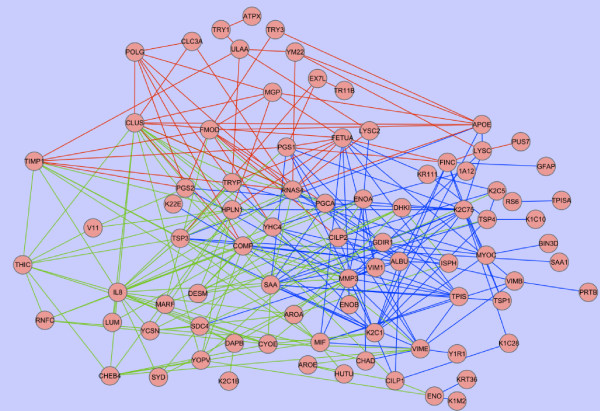
**Protein interaction network generated from the top 100 BioHEL protein pairs for the emPAI canine articular cartilage dataset.** The most frequently used protein pairs for IL-1β in blue, carprofen in red and carprofen + IL-1β in green.

The interaction network generated from the ProteinProphet probabilities is shown in Figure 3. There are particular proteins (those most connected to other proteins) that can be identified from the network. These proteins include matrix metalloproteinase-3 (MMP-3), interleukin 8 (IL-8), HPLN1, matrix gla protein (MGP) and APOE, and are detailed in Table 6. The interaction network generated from the emPAI scores is shown in Figure 4. In this network there are fewer highly connected proteins, than in the ProteinProphet network, although MMP-3 and IL-8 are again connected to many other proteins. The fewer highly connected proteins in the emPAI network could be due to some proteins having similar emPAI scores but differing ProteinProphet probabilities. Therefore, where in the ProteinProphet network only one protein was suitable, in the emPAI network multiple proteins gave the same results and were interchangeable.

**Table 6:** Most connected proteins identified from the ProteinProphet protein pairs network

**Most connected proteins**	**Description**
interleukin 8 (IL-8)	A chemotactic factor known to attract neutrophils, basophils, and T-cells and is involved in neutrophil activation. IL-8 is released from a number of cell types in response to an inflammatory stimulus [46].
Matrix metalloproteinase-3 (MMP-3)	MMP-3 can degrade fibronectin, laminin, some gelatins, various collagens and cartilage proteoglycans [47,48].
Apolipoprotein E (APOE)	APOE mediates the binding, internalization, and catabolism of lipoprotein particles [49,50].
Matrix gla protein (MGP)	MGP interacts with the matrix of bone and cartilage and is thought to act as an inhibitor of bone formation [51,52].
Hyaluronan and proteoglycan link protein 1 (HPLN1)	HPLN1 stabilizes the aggregates of proteoglycan monomers with hyaluronic acid in the extracellular cartilage matrix [53].

COMP is a noncollagenous ECM protein that is abundantly expressed in articular cartilage and which has been considered by other groups as a possible marker of articular cartilage degradation. This protein was not included in any top ranking protein lists, or in either network generated, because COMP was found at similar levels across all samples, regardless of the type of treatment. Therefore the bioinformatics methods discussed here are useful in determining proteins that may be suitable for use as putative biomarkers, rather than simply proteins that are abundant. We also expected to detect MMPs, a family of proteins expressed by chondrocytes with roles in cartilage development, remodelling and disease [54]. Matrix metalloproteinase-3 (MMP-3), a surrogate biomarker of psoriatic and rheumatoid arthritis [55,56], was pivotal in the classification of IL-1β samples. MMP-3 is a proteolytic enzyme known to degrade components of the ECM, including collagens and cartilage proteoglycans and, as a result, was the top ranking protein for the IL-1β class. No other MMPs were highlighted by the bioinformatics techniques applied. interleukin 8 (IL-8) was dominant in the classification of IL-1β and carprofen treated samples. IL-8 is the major chemotactic factor released in response to pro-inflammatory cytokines in synovial tissues from rheumatoid arthritis and osteoarthritis affected joints [57-59]. Matrix gla protein, involved in inhibition of calcification in cartilage [51], was also frequently found in the BioHEL rules from the analysis of the ProteinProhet dataset. This protein was found in many samples across all treatment groups, except for the carprofen + IL-1β group. MMP-3, IL-8 and MGP were also the most connected proteins in the ProteinProphet network. The inclusion of proteins such as these in the top ranking lists and as the most connected proteins, demonstrates the abilities of these techniques aimed at identifying proteins involved in cartilage degradation. There were other proteins, such as APOE and HPLN1 that were found frequently in the rules. However, the supplementary tables show they are not present in all the samples of any group.

The proteins identified by this protocol were compared to those found using the same proteomics protocol, but without the bioinformatics analysis, using equine explant tissue [33]. There were proteins highlighted in the equine study that were not in this canine study, including COMP, fibronectin and chondroadherin, because, whilst they were abundant in the samples, they were not significantly different across the different treatment groups. Therefore, the bioinformatics methods used provide a way to focus on the most relevant proteins.

The data indicate that in the absence of IL-1β carprofen had little effect on the cartilage explant secretome. Therefore, proteins that aided in the classification may have been included in the classification model, but are not necessarily intrinsically involved in the processes being investigated. This resulted in some non-mammalian proteins identified as top ranking proteins. It is possible traces of contaminating proteins entered the analysis and the proteins have been correctly identified. Alternatively, proteins were incorrectly identified by Mascot; because the selected proteins were not in the database used, in which case the highest-ranking closest protein was used.

The major challenge faced by many proteomic studies is the under representation of the lower abundance proteins that are potentially of interest. This under representation is due to the massive range of protein abundance in complex biological samples such as serum, cerebrospinal fluid and urine or marginally less complex samples like the secretome [60] with high abundant proteins saturating the MS/MS with higher signal levels. Proteins, such as COMP, are highly abundant in the cartilage and hinder identification of less abundant proteins relevant to biological processes. Sample preparation techniques such as proteome fractionation and deglycosylation should enable the identification of less abundant proteins and therefore more information could be uncovered using these techniques.

As described, additional analyses were performed on a number of top ranking proteins identified by these methods. However, further analysis is required to investigate the individual proteins highlighted and other proteins in the networks. This includes both laboratory-based experiments to confirm the presence of individual proteins and their levels within different sample types, and further literature and pathway analyses to mine relevant previously identified information.

Due to the nature of the machine learning methods used, it would be more suitable to analyze larger datasets and therefore future work should include a similar study on a larger scale, with more replicate samples and a larger number of animals.

## Conclusions

This study involved bioinformatic analysis of high throughput proteomic data generated using an explant model of cytokine-induced articular cartilage degradation. The approach described in this paper may be used in future studies for identification of early structural changes in cartilage and for drug testing, and screening of novel anti-inflammatory compounds from natural products. Extending our previous work with explant models of articular cartilage, bioinformatics techniques were applied to high throughput proteomics data to identify proteins suitable for use as exploratory biomarkers. This combination of laboratory-based and computational methods has provided results, which experimental techniques alone could not have provided. This proteomic and bioinformatics study has detected a number of established ECM proteins, including MMP-3, IL-8 and MGP, and therefore has shown the application of these bioinformatics tools is suitable for this purpose and could be applied to proteomics data from other areas.

### Endnotes

^a^http://www.legislation.gov.uk/ukpga/1986/14/contents

^b^http://www.legislation.gov.uk/ukpga/1966/36

## Abbreviations

APOE: Apolipoprotein E; COMP: Cartilage oligomeric matrix protein; DMEM: Dulbecco’s modified eagle medium; DTT: Dithiothreitol; ECM: Extracellular matrix; emPAI: Exponentially modified protein abundance index; HPLC: High-performance liquid chromatography; HPLN1: Hyaluronan and proteoglycan link protein 1; IL-1β: Interleukin 1 beta; IL-8: Interleukin-8; MGP: Matrix gla protein; MMP: Matrix metalloproteinase; MMP-3: Matrix metalloproteinase-3 (stromelysin-1); MS: Mass spectrometry; OA: Osteoarthritis.

## Competing interests

This study has received industrial grant support from Mars^®^ and WALTHAM^®^. The funding provided supported Kirsty L Hillier (through a one-year M.Res. studentship) and supplemented the BBSRC Industrial CASE Ph.D. studentship awarded to Anna L. Swan.

## Authors’ information

Jaume Bacardit and Ali Mobasheri

The D-BOARD European Consortium for Biomarker Discovery, University of Nottingham, University Park, Nottingham, NG7 2RD, United Kingdom;

http://cordis.europa.eu/projects/rcn/105314_en.html

http://ec.europa.eu/research/health/medical-research/severe-chronic-diseases/projects/d-board_en.html

## Pre-publication history

The pre-publication history for this paper can be accessed here:

http://www.biomedcentral.com/1471-2474/14/349/prepub

## Supplementary Material

Additional file 1: Table S1 Proteins identified by Mascot in the control (untreated) samples with corresponding Mascot scores. The Mascot score is a probability based score, used to determine the significance of a protein match. The higher the score the less likely it is that the protein match occurred by random. Click here for file

Additional file 2: Table S2 Proteins identified by Mascot in the IL-1β treated samples with their corresponding Mascot scores. Click here for file

Additional file 3: Table S3 Proteins identified by Mascot in the carprofen treated samples with their corresponding Mascot scores. Click here for file

Additional file 4: Table S4 Proteins identified by Mascot in the samples treated with a combination of carprofen and IL-1β and their corresponding Mascot scores. Click here for file

Additional file 5: Figure S1 SDS-PAGE protein profile of secretome from dog one. a) control (1,2,3,4), IL-1β (5,6,7,8) b) control (1,2,3,4), carprofen (5,6,7,8) c) control (1,2,3,4), IL-1β + carprofen (5,6,7,8). Molecular weight markers (M) (in kDa) were Bio-Rad Precision Plus unstained standards. Click here for file

Additional file 6: Figure S2 SDS-PAGE protein profile of secretome from dog two. a) control (1,2,3,4), IL-1β (5,6,7,8) b) control (1,2,3,4), carprofen (5,6,7,8) c) control (1,2,3,4), IL-1β + carprofen (5,6,7,8). Molecular weight markers (M) (in kDa) were Bio-Rad Precision Plus unstained standards. Lanes 1 – 8 each contain 14-μg protein. Lane 9 contains blank loading buffer control. Arrows indicate differences in protein bands between sample sets. Gels were silver stained. Click here for file
