# Supplementary material for: Analysis of mass spectrometry data from the secretome of an explant model of articular cartilage exposed to pro-inflammatory and anti-inflammatory stimuli using machine learning
Source: BMC Musculoskelet Disord. 2013 Dec 13;14:349. doi: 10.1186/1471-2474-14-349 (PMC3878677; doi:10.1186/1471-2474-14-349)
Supplement: Additional file 1: Table S1 — Proteins identified by Mascot in the control (untreated) samples with corresponding Mascot scores. The Mascot score is a probability based score, used to determine the significance of a protein match. The higher the score the less likely it is that the protein match occurred by random. [file 1471-2474-14-349-S1.doc]

Additional file 1: Table S1. Proteins identified by Mascot in the control (untreated) samples with corresponding Mascot scores. The Mascot score is a probability based score, used to determine the significance of a protein match. The higher the score the less likely it is that the protein match occurred by random.

| Protein | SwissProt accession number | Control Sample 1 | Control Sample 2 | Control Sample 3 | Control Sample 4 | Control Sample 5 | Control Sample 6 |
| --- | --- | --- | --- | --- | --- | --- | --- |
| Cartilage oligomeric matrix protein | Q9R0G6 | 678 | 740 | 703 | 935 | 722 | 732 |
| Aggrecan core protein | **Q28343** | 492 | 713 | 452 | 1170 | 673 | 374 |
| Fibronectin | **P07589** | 316 | 321 | 207 | 334 | 196 | 272 |
| Clusterin | **P25473** | 167 | 180 | 220 | 97 | 111 | 159 |
| Decorin | **Q29393** | 158 | 205 | 158 | 143 | 117 | 124 |
| Chondroadherin | **O15335** | 140 | 209 | 201 | 242 | 147 | 282 |
| Trypsin | **P00761** | 109 | 148 | 173 | 150 | 102 | 180 |
| Keratin, type II cytoskeletal 1 | **P04264** | 90 | - | - | - | - | 51 |
| Biglycan | **O02678** | 75 | 75 | 55 | - | 64 | 79 |
| Anionic trypsin-1 | **P00762** | 56 | 72 | 80 | 108 | 63 | 111 |
| Keratin, type II cytoskeletal 2 | **P35908** | 56 | - | - | - | - | - |
| Thrombospondin-3 | **P49746** | 51 | 50 | 51 | - | 56 | 60 |
| Keratin, type II cytoskeletal 79 | **Q148H7** | 51 | - | - | - | - | - |
| Putative heat shock protein 2 | **P85917** | 51 | - | - | - | - | - |
| Fibromodulin | **Q06828** | 46 | - | - | - | - | - |
| Ribonuclease 4 | **P15467** | 45 | 44 | 63 | 43 | 34 | 38 |
| Matrix Gla protein | **P08493** | 42 | 47 | 62 | - | 37 | 63 |
| Vimentin | **P48673** | 42 | - | - | - | - | - |
| Lactadherin | **P21956** | 31 | - | - | - | - | - |
| Myeloid differentiation primary response protein MyD88 | **A5HNF6** | 19 | - | - | - | - | - |
| Uncharacterized protein MJ0726 | **Q58136** | 19 | - | - | - | - | - |
| Metalloproteinase inhibitor 1 | **P81546** | - | 67 | - | 70 | - | 76 |
| Uncharacterized endonuclease C19F8.04c | **O60168** | - | 66 | 62 | 50 | - | 53 |
| Lysozyme C, spleen isozyme | **P81709** | - | 60 | - | - | - | 41 |
| Eukaryotic translation initiation factor 3 subunit J | **Q8I1G8** | - | 51 | - | - | - | - |
| Fibritin | P10104 | - | 50 | - | - | - | - |
| Alpha-1-antitrypsin | O00394 | - | 46 | - | - | - | 44 |
| Cationic trypsin-3 | P08426 | - | 29 | - | - | - | - |
| Hyaluronan and proteoglycan link protein 1 | **P55252** | - | - | 57 | - | 56 | - |
| Anthranilate synthase component 1 | **Q06128** | - | - | 49 | - | - | - |
| Lysozyme C, spleen isozyme | **P81709** | - | - | 41 | 42 | - | - |
| Nucleoprotein | **P18448** | - | - | 39 | - | - | - |
| Cartilage intermediate layer protein 1 | **Q66K08** | - | - | 33 | - | - | - |
| Erythronate-4-phosphate dehydrogenase | **A6VXM3** | - | - | 32 | - | - | - |
| Transcription cofactor vestigial-like protein 1 | **Q99NC0** | - | - | 30 | - | - | - |
| Gene D protein | **P10312** | - | - | 26 | - | - | - |
| Thrombospondin-4 | **P35443** | - | - | - | 241 | 218 | 60 |
| C-type lectin domain family 3 member A | **Q9EPW4** | - | - | - | 58 | 56 | 58 |
| Protoheme IX farnesyltransferase | **C1DG34** | - | - | - | 55 | - | - |
| Chemotaxis response regulator protein-glutamate methylesterase 4 | **Q39S45** | - | - | - | 55 | - | - |
| Phosphoribosylformylglycinamidine synthase 2 | **A7H373** | - | - | - | 50 | - | 41 |
| Cartilage intermediate layer protein 2 | **Q8IUL8** | - | - | - | 44 | - | 44 |
| Target of Nesh-SH3 | **Q7Z7G0** | - | - | - | 41 | - | - |
| Apolipoprotein E | **P18649** | - | - | - | 40 | - | - |
| Probably inactive leucine-rich repeat receptor-like protein kinase At1g50610 | **Q9LPT1** | - | - | - | 40 | - | - |
| Aspartyl-tRNA synthetase | **A1KAJ9** | - | - | - | 35 | - | - |
| Dihydrodipicolinate reductase | **A1S8K1** | - | - | - | 35 | - | - |
| Uncharacterized oxidoreductase ycsN | **P42972** | - | - | - | 35 | - | - |
| Transmembrane GTPase Marf | **Q7YU24** | - | - | - | 35 | - | - |
| Probable 26S proteasome non-ATPase regulatory subunit 3 | **P25161** | - | - | - | 34 | - | - |
| Glucose-6-phosphate isomerase | **A0L900** | - | - | - | 27 | - | - |
| Glutamyl-tRNA synthetase | **Q2NIP6** | - | - | - | - | 54 | - |
| Keratin, type I cytoskeletal 9 | **P35527** | - | - | - | - | 36 | - |
| Catalase | **Q27710** | - | - | - | - | 36 | - |
| 30S ribosomal protein S2 | **A5G7W8** | - | - | - | - | 35 | - |
| Pre-rRNA-processing protein pro-1 | **Q22006** | - | - | - | - | 32 | - |
| NHS-like protein 1 | **Q8CAF4** | - | - | - | - | - | 56 |
| Keratin, type II cytoskeletal 75 | **O60168** | - | - | - | - | - | 53 |
| SPARC | **P13213** | - | - | - | - | - | 48 |
| Cadherin-1 | **P12830** | - | - | - | - | - | 35 |
| IAA-amino acid hydrolase ILR1-like 3 | **Q851L5** | - | - | - | - | - | 34 |
| 3-phosphoshikimate 1-carboxyvinyltransferase | **Q6D401** | - | - | - | - | - | 34 |
| Shikimate | **Q1AWA3** | - | - | - | - | - | 34 |
