# Supplementary material for: Analysis of mass spectrometry data from the secretome of an explant model of articular cartilage exposed to pro-inflammatory and anti-inflammatory stimuli using machine learning
Source: BMC Musculoskelet Disord. 2013 Dec 13;14:349. doi: 10.1186/1471-2474-14-349 (PMC3878677; doi:10.1186/1471-2474-14-349)
Supplement: Additional file 2: Table S2 — Proteins identified by Mascot in the IL-1β treated samples with their corresponding Mascot scores. [file 1471-2474-14-349-S2.doc]

Additional file 2: Table S1. Proteins identified by Mascot in the IL-1β treated samples with their corresponding Mascot scores.

| Protein | SwissProt accession number | IL1β Sample 1 | IL1β Sample 2 | IL1β Sample 3 | IL1β Sample 4 | IL1β Sample 5 | IL1β Sample 6 |
| --- | --- | --- | --- | --- | --- | --- | --- |
| Cartilage oligomeric matrix protein | **Q9R0G6** | 1006 | 958 | 795 | 821 | 1048 | 821 |
| Aggrecan core protein | **Q28343** | 1170 | 639 | 642 | 529 | 630 | 448 |
| Fibronectin | **Q28275** | 175 | 254 | 173 | 166 | 255 | 116 |
| Clusterin | **P25473** | 73 | 59 | 95 | 53 | 65 | 51 |
| Decorin | **Q29393** | 250 | 96 | 129 | 117 | 163 | 131 |
| Chondroadherin | **O15335** | 226 | 197 | 156 | 117 | 156 | 151 |
| Trypsin | **P00761** | 156 | 82 | 117 | 109 | 127 | 104 |
| Keratin, type II cytoskeletal 1 | **A5A6M6** | 67 | - | - | - | 218 | 120 |
| Biglycan | **O02678** | 65 | 55 | 65 | 56 | 74 | - |
| Anionic trypsin-1 | **P00762** | 81 | 60 | 80 | 76 | 75 | 72 |
| Keratin, type II cytoskeletal 2 | **P35908** | - | - | - | - | - | 75 |
| Thrombospondin-3 | **P49746** | 49 | - | 57 | - | 53 | - |
| Ribonuclease 4 | **P15467** | 56 | 37 | 51 | - | 40 | 43 |
| Matrix Gla protein | **P08493** | 49 | - | - | - | 67 | 48 |
| Vimentin | **P48616** | - | 67 | 63 | 131 | 58 | 33 |
| Uncharacterized endonuclease C19F8.04c | **O60168** | - | 65 | - | - | - | 44 |
| Lysozyme C, spleen isozyme | **P81709** | 99 | 90 | - | - | - | 30 |
| Cationic trypsin-3 | **P08426** | - | - | - | - | - | 27 |
| Hyaluronan and proteoglycan link protein 1 | **P55252** | - | - | - | 55 | 51 | - |
| Cartilage intermediate layer protein 1 | **O19112** | 40 | 91 | - | 38 | 26 | - |
| Thrombospondin-4 | **P35443** | - | 178 | 242 | 259 | - | - |
| Cartilage intermediate layer protein 2 | **Q8IUL8** | 79 | 55 | 39 | 65 | 59 | - |
| Apolipoprotein E | P18649 | 63 | - | - | - | - | - |
| Keratin, type I cytoskeletal 9 | **P35527** | - | - | - | - | - | 35 |
| Keratin, type II cytoskeletal 75 | **Q08D91** | 67 | - | - | - | - | 67 |
| Stromelysin-1 | **Q6Y4Q5** | 89 | 77 | 95 | 100 | 87 | 166 |
| Thrombospondin-1 | **P07996** | 85 | - | 97 | - | 57 | 54 |
| Lysozyme C | **P79847** | 83 | 50 | - | - | - | - |
| Alpha-enolase | **P17182** | 69 | 110 | 102 | 94 | - | 83 |
| Beta-enolase | **Q3ZC09** | 69 | 110 | - | - | - | 79 |
| Serum albumin | **P02769** | 53 | - | 44 | - | 33 | - |
| Phosphomethylpyrimidine synthase | **C0QJ00** | 52 | - | - | - | - | - |
| Serum amyloid A protein | **P35541** | 47 | - | 45 | 38 | 51 | 41 |
| Myocilin | **Q2PT31** | 47 | - | - | - | - | - |
| Triadin | **Q13061** | 39 | - | - | - | - | - |
| Syndecan-4 | **Q8HZJ6** | 38 | - | - | - | - | - |
| Probable methyltransferase Y17G7B.18 | **Q9U2R0** | 31 | - | - | - | - | - |
| Keratin, type I microfibrillar 48 kDa, component 8C-1 | **P02534** | - | 112 | - | - | - | - |
| Keratin, type II cuticular Hb1 | **Q148H4** | - | 106 | - | - | - | - |
| Keratin, type I microfibrillar, 47.6 kDa | **P25690** | - | 101 | - | - | - | - |
| Alpha-2-HS-glycoprotein | **P12763** | - | 80 | - | 42 | - | 52 |
| Keratin, type II microfibrillar, component 7C | **P15241** | - | 73 | - | - | - | - |
| Keratin, type II cytoskeletal 5 | **Q5XQN5** | - | 70 | 62 | - | - | - |
| Keratin, type II microfibrillar, component 5 | **P25691** | - | 67 | - | - | - | - |
| Keratin, type I cuticular Ha6 | **O76013** | - | 65 | - | - | - | - |
| Chaperone protein mrkB | **P21646** | - | 52 | - | - | - | - |
| ATP-dependent protease ATPase subunit HslU | **C5A098** | - | 50 | - | - | - | - |
| Rho GTPase-activating protein 31 | **A6X8Z5** | - | 48 | - | - | - | - |
| Vimentin-1/2 | **P24789** | - | 42 | - | 76 | - | 33 |
| Keratin, type I cuticular Ha5 | **Q497I4** | - | 42 | - | - | - | - |
| Uncharacterized 50 kDa protein in type I retrotransposable element R1DM | **P16424** | - | 40 | - | - | - | - |
| Protein dfrA | **O52178** | - | 39 | - | - | - | - |
| UPF0133 protein Cphy_0047 | **A9KQC8** | - | 34 | - | - | - | - |
| N-acetyl-gamma-glutamyl-phosphate reductase | **A6L849** | - | 34 | - | - | - | - |
| Keratin-associated protein 11-1 | **Q6R648** | - | 31 | - | - | - | - |
| Enolase | **P56252** | - | 30 | 85 | 94 | - | - |
| Extracellular fatty acid-binding protein | **P21760** | - | 28 | - | - | - | - |
| Triosephosphate isomerase | **P54714** | - | - | 68 | 42 | - | 73 |
| Macrophage migration inhibitory factor | **P14174** | - | - | 47 | 46 | - | 64 |
| DNA-binding protein SATB1 | **Q01826** | - | - | 37 | - | - | - |
| Serum amyloid A-1 protein | **P20726** | - | - | 33 | - | - | - |
| Phosphatidylethanolamine-binding protein 1 | **P13696** | - | - | 30 | - | - | - |
| ATP-dependent Clp protease ATP-binding subunit ClpX | **B0C146** | - | - | 29 | - | - | - |
| 50S ribosomal protein L33 1 | **P78015** | - | - | 25 | - | - | - |
| Interleukin-8 | **P41324** | - | - | - | 54 | - | - |
| Glial fibrillary acidic protein | **Q58EE9** | - | - | - | 41 | - | - |
| Protein S100-A1 | **P02639** | - | - | - | 36 | - | - |
| Streptogrisin-B | **P00777** | - | - | - | 35 | - | - |
| 1-aminocyclopropane-1-carboxylate synthase 2 | **Q00379** | - | - | - | 28 | - | - |
| GTP-binding protein lepA | **Q92BN4** | - | - | - | 27 | - | - |
| Keratin, type I cytoskeletal 10 | **P13645** | - | - | - | - | 135 | - |
| Keratin, type II cytoskeletal 1b | **Q6IFZ6** | - | - | - | - | 83 | 46 |
| Keratin, type II cytoskeletal 73 | **Q6NXH9** | - | - | - | - | 57 | - |
| 4-hydroxy-3-methylbut-2-enyl diphosphate reductase | **A1JJE4** | - | - | - | - | 50 | - |
| Rho GDP-dissociation inhibitor 1 | **P19803** | - | - | - | - | 21 | - |
| Keratin, type II cytoskeletal 6B | **P04259** | - | - | - | - | - | 75 |
| ATP-dependent RNA helicase DBP8 | **Q756G5** | - | - | - | - | - | 61 |
| Keratin, type I cytoskeletal 16 | **P08779** | - | - | - | - | - | 58 |
| Keratin, type I cytoskeletal 19 | **P86246** | - | - | - | - | - | 58 |
| Triosephosphate isomerase A | **Q1MTI4** | - | - | - | - | - | 54 |
| 14-3-3 protein epsilon | **P62261** | - | - | - | - | - | 48 |
| 30S ribosomal protein S6 | **Q30PZ4** | - | - | - | - | - | 48 |
| Nuclear receptor subfamily 1 group I member 3 | **P62044** | - | - | - | - | - | 27 |
| Bifunctional protein folD | **Q48SM7** | - | - | - | - | - | 23 |
