# Supplementary material for: Analysis of mass spectrometry data from the secretome of an explant model of articular cartilage exposed to pro-inflammatory and anti-inflammatory stimuli using machine learning
Source: BMC Musculoskelet Disord. 2013 Dec 13;14:349. doi: 10.1186/1471-2474-14-349 (PMC3878677; doi:10.1186/1471-2474-14-349)
Supplement: Additional file 3: Table S3 — Proteins identified by Mascot in the carprofen treated samples with their corresponding Mascot scores. [file 1471-2474-14-349-S3.doc]

Additional file 3: Table S1. Proteins identified by Mascot in the carprofen treated samples with their corresponding Mascot scores.

| Protein | SwissProt accession number | Carprofen Sample 1 | Carprofen Sample 2 | Carprofen Sample 3 | Carprofen Sample 4 | Carprofen Sample 5 | Carprofen Sample 6 |
| --- | --- | --- | --- | --- | --- | --- | --- |
| Cartilage oligomeric matrix protein | **Q9R0G6** | 738 | 936 | 638 | 481 | 797 | 422 |
| Aggrecan core protein | **Q28343** | 502 | 638 | 297 | 188 | 516 | 174 |
| Fibronectin | **Q28275** | 304 | 227 | 170 | 115 | 124 | 156 |
| Clusterin | **P25473** | 207 | 107 | 211 | 158 | 140 | 204 |
| Decorin | **Q29393** | 177 | 141 | 168 | 106 | 136 | 108 |
| Chondroadherin | **O15335** | 174 | 155 | 94 | 112 | 152 | 62 |
| Trypsin | **P00761** | 118 | 120 | 83 | 99 | 112 | 164 |
| Biglycan | **O02678** | 60 | 83 | 57 | 62 | 58 | 60 |
| Anionic trypsin-1 | **P00762** | 86 | 64 | 69 | 73 | 69 | 78 |
| Thrombospondin-3 | **P49746** | 56 | 43 | - | - | 46 | 56 |
| Fibromodulin | **P50608** | - | - | 56 | 52 | 44 | - |
| Ribonuclease 4 | **P15467** | 54 | 42 | - | 44 | 46 | 36 |
| Matrix Gla protein | **P08493** | 40 | 42 | 42 | 40 | 71 | 45 |
| Metalloproteinase inhibitor 1 | **P81546** | - | - | 41 | - | 46 | - |
| Uncharacterized endonuclease C19F8.04c | **O60168** | - | 60 | - | 49 | 55 | - |
| Lysozyme C, spleen isozyme | **P81709** | 62 | 76 | - | - | 55 | 35 |
| Cationic trypsin-3 | **P08426** | - | - | - | - | 31 | - |
| Hyaluronan and proteoglycan link protein 1 | **P55252** | 54 | 52 | - | 44 | 61 | - |
| Cartilage intermediate layer protein 1 | **O19112** | 35 | 27 | - | - | 38 | - |
| Thrombospondin-4 | **Q3SWW8** | - | 249 | - | 189 | - | 198 |
| C-type lectin domain family 3 member A | **Q9EPW4** | 61 | - | 77 | - | - | - |
| Protoheme IX farnesyltransferase | **C1DG34** | - | 50 | - | - | - | - |
| Cartilage intermediate layer protein 2 | **Q8IUL8** | - | - | - | - | 64 | - |
| Apolipoprotein E | **P18649** | - | 38 | 40 | - | 53 | - |
| NHS-like protein 1 | **Q8CAF4** | - | 47 | - | - | - | - |
| SPARC | **P13213** | - | - | - | 39 | - | - |
| Lysozyme C | **P79847** | 31 | - | - | - | 55 | - |
| Myocilin | **Q2PT31** | - | - | 54 | - | - | - |
| Alpha-2-HS-glycoprotein | **P12763** | - | 34 | - | 49 | - | - |
| Genome polyprotein | **P09866** | 51 | - | 30 | - | - | - |
| Chaperone protein htpG | **Q47XA7** | 51 | - | - | - | - | - |
| Ribulose bisphosphate carboxylase | **Q2RRP5** | 36 | - | - | - | - | - |
| Prolargin | **Q9JK53** | 32 | - | - | - | - | - |
| UPF0284 protein Pcal_1534 | **A3MWD6** | 28 | - | - | - | - | - |
| Arginyl-tRNA synthetase | **Q72CN5** | 25 | - | - | - | - | - |
| Chemotaxis response regulator protein-glutamate methylesterase 4 | **Q39S45** | - | 50 | - | - | - | - |
| Putative malate dehydrogenase 1B | **A3KMX7** | - | 41 | - | - | - | - |
| Uncharacterized protein YMR144W | **P40214** | - | 40 | - | - | 40 | - |
| Formin-1 | **Q68DA7** | - | 34 | - | - | - | - |
| Ribosome maturation factor rimP | **Q65JI5** | - | 28 | - | - | - | - |
| Ribonucleoside-diphosphate reductase nrdZ | **O53767** | - | - | 54 | - | - | - |
| Histidyl-tRNA synthetase | **Q6KI17** | - | - | 30 | - | - | - |
| Outer capsid protein VP4 | **Q01641** | - | - | 30 | - | - | - |
| Fc receptor-like A | **Q920A9** | - | - | 30 | - | - | - |
| Protein grpE | **Q03WI1** | - | - | 30 | - | - | - |
| Probable inactive receptor-like kinase SSP | **Q7XJT7** | - | - | 30 | - | - | - |
| 2~,3~-cyclic-nucleotide 2~-phosphodiesterase | **Q3B4Y9** | - | - | 30 | - | - | - |
| Chromobox protein homolog 2 | **Q14781** | - | - | - | 38 | - | - |
| Girdin | **Q3V6T2** | - | - | - | 36 | - | - |
| 1-deoxy-D-xylulose 5-phosphate reductoisomerase | **B1YI70** | - | - | - | 27 | - | - |
| Acetyl-coenzyme A carboxylase carboxyl transferase subunit beta | **Q97DB1** | - | - | - | 24 | - | - |
| Alanyl-tRNA synthetase | **A6LEZ8** | - | - | - | - | 51 | - |
| Tumor necrosis factor receptor superfamily member 11B | **O00300** | - | - | - | - | 50 | - |
| Methylthioribose-1-phosphate isomerase | **B2VZQ8** | - | - | - | - | 47 | - |
| Exodeoxyribonuclease 7 large subunit | **B2SAR1** | - | - | - | - | 41 | - |
| Ascorbate-specific permease IIC component ulaA | **P75291** | - | - | - | - | 33 | - |
| RNA pseudourine synthase 7 | **Q0E0Y3** | - | - | - | - | 22 | - |
| D-(-)-3-hydroxybutyrate oligomer hydrolase | **Q13Q76** | - | - | - | - | - | 29 |
| Bifunctional endo-1,4-beta-xylanase xylA | **P29126** | - | - | - | - | - | 29 |
