# Supplementary material for: Analysis of mass spectrometry data from the secretome of an explant model of articular cartilage exposed to pro-inflammatory and anti-inflammatory stimuli using machine learning
Source: BMC Musculoskelet Disord. 2013 Dec 13;14:349. doi: 10.1186/1471-2474-14-349 (PMC3878677; doi:10.1186/1471-2474-14-349)
Supplement: Additional file 4: Table S4 — Proteins identified by Mascot in the samples treated with a combination of carprofen and IL-1β and their corresponding Mascot scores. [file 1471-2474-14-349-S4.doc]

Additional file 4: Table S1. Proteins identified by Mascot in the samples treated with a combination of carprofen and IL-1β and their corresponding Mascot scores.

| Protein | SwissProt accession number | Carprofen + IL1β Sample 1 | Carprofen + IL1β Sample 2 | Carprofen + IL1β Sample 3 | Carprofen + IL1β Sample 4 | Carprofen + IL1β Sample 5 |
| --- | --- | --- | --- | --- | --- | --- |
| Cartilage oligomeric matrix protein | **Q9R0G6** | 901 | 1234 | 1117 | 1163 | 1078 |
| Aggrecan core protein | **Q28343** | 611 | 665 | 680 | 943 | 677 |
| Fibronectin | **Q28275** | 309 | 215 | 415 | 339 | 237 |
| Clusterin | **P25473** | 169 | 128 | 127 | 72 | 108 |
| Decorin | **Q29393** | 117 | 124 | 153 | 143 | 97 |
| Chondroadherin | **O15335** | 64 | 76 | 174 | 134 | 194 |
| Trypsin | **P00761** | 129 | 107 | 103 | 120 | 71 |
| Keratin, type II cytoskeletal 1 | **A5A6M6** | 64 | 73 | - | - | 43 |
| Biglycan | **O02678** | 68 | 71 | - | 64 | 58 |
| Anionic trypsin-1 | **P00762** | 70 | 67 | 60 | 69 | 51 |
| Thrombospondin-3 | **P49746** | 50 | 57 | 54 | - | - |
| Fibromodulin | **P50608** | 43 | - | - | 49 | - |
| Ribonuclease 4 | **P15467** | 37 | 49 | 43 | - | 56 |
| Vimentin | **P48616** | - | 64 | 54 | - | 75 |
| Metalloproteinase inhibitor 1 | **P81546** | - | 41 | - | - | - |
| Uncharacterized endonuclease C19F8.04c | **O60168** | 48 | - | - | 62 | 58 |
| Hyaluronan and proteoglycan link protein 1 | **P55252** | - | 43 | - | 37 | - |
| Cartilage intermediate layer protein 1 | **O19112** | - | 42 | 40 | 41 | 56 |
| Thrombospondin-4 | **Q3SWW8** | - | 60 | 54 | - | - |
| C-type lectin domain family 3 member A | **Q9EPW4** | - | - | 48 | - | - |
| Protoheme IX farnesyltransferase | **C1DG34** | - | 50 | - | 50 | - |
| Chemotaxis response regulator protein-glutamate methylesterase 4 | **Q39S45** | - | 50 | - | 50 | - |
| Cartilage intermediate layer protein 2 | **Q8IUL8** | - | 75 | 60 | 37 | 48 |
| Aspartyl-tRNA synthetase | **A1KAJ9** | 34 | - | - | 36 | - |
| Dihydrodipicolinate reductase | **A1S8K1** | 34 | - | - | 36 | - |
| Uncharacterized oxidoreductase ycsN | **P42972** | 34 | - | - | 36 | - |
| Transmembrane GTPase Marf | **Q7YU24** | 34 | - | - | 35 | - |
| IAA-amino acid hydrolase ILR1-like 3 | **Q851L5** | - | - | - | 32 | - |
| Stromelysin-1 | **Q6Y4Q5** | 139 | 94 | 104 | 73 | - |
| Thrombospondin-1 | **P35441** | 64 | - | 85 | - | - |
| Alpha-enolase | **Q9PVK2** | 83 | - | - | - | - |
| Phosphomethylpyrimidine synthase | **C0QJ00** | - | 49 | 53 | - | - |
| Serum amyloid A protein | **P35541** | 53 | 48 | 54 | - | - |
| Syndecan-4 | **P31431** | 47 | - | - | 26 | - |
| Alpha-2-HS-glycoprotein | **P12763** | - | - | - | - | 32 |
| Keratin, type II cytoskeletal 5 | **Q5XQN5** | 67 | - | - | - | - |
| Enolase | **O02654** | - | 73 | - | - | - |
| Macrophage migration inhibitory factor | **P14174** | - | 52 | - | - | - |
| Interleukin-8 | **P41324** | 44 | 44 | 58 | 42 | 61 |
| Keratin, type II cytoskeletal 1b | **Q6IG01** | 24 | - | - | - | - |
| 4-hydroxy-3-methylbut-2-enyl diphosphate reductase | **A1JJE4** | - | - | - | 72 | - |
| Genome polyprotein | **Q6YMS3** | - | - | - | - | 16 |
| Prolargin | **Q9JK53** | 39 | - | - | - | - |
| Lumican | **P51884** | 43 | - | 44 | 41 | - |
| Centrosome-associated protein 350 | **Q5VT06** | 39 | - | - | - | - |
| Desmin | **O62654** | - | 49 | - | - | - |
| Electron transport complex protein rnfC | **Q5PIB9** | - | 28 | 26 | - | - |
| Uncharacterized protein V11 | **B4YNF1** | - | 26 | - | - | - |
| SPBc2 prophage-derived uncharacterized protein yopV | **O34869** | - | 22 | - | - | - |
| Single-stranded DNA-binding protein | **Q8G757** | - | - | 31 | - | - |
| Vacuolar protein-sorting protein BRO1 | **Q6BRL3** | - | - | 30 | - | - |
| Protein EFR3 | **Q4I1T9** | - | - | 27 | - | - |
| Urocanate hydratase | **A7ZAE5** | - | - | - | 38 | - |
| OS=Erwinia carotovora subsp. atroseptica GN=aroA PE=3 SV=1 | **Q6D401** | - | - | - | 32 | - |
| Shikimate dehydrogenase | **Q1AWA3** | - | - | - | 32 | - |
| Hybrid signal transduction histidine kinase I | **Q86AT9** | - | - | - | 24 | - |
| Forkhead-associated domain-containing protein 1 | **A6PWD2** | - | - | - | - | 36 |
| Uncharacterized 17.7 kDa protein in e-segB intergenic region | **P32271** | - | - | - | - | 27 |
| Probable LRR receptor-like serine/threonine-protein kinase At2g02780 | **C0LGJ9** | - | - | - | - | 24 |
| Serine/threonine-protein kinase STE20 | **Q03497** | - | - | - | - | 16 |
